# Cellulose Acetate Microbeads for Controlled Delivery of Essential Micronutrients

**DOI:** 10.1021/acssuschemeng.2c07269

**Published:** 2023-03-14

**Authors:** Ciarán Callaghan, Davide Califano, Marcos Henrique Feresin Gomes, Hudson Wallace Pereira de Carvalho, Karen J. Edler, Davide Mattia

**Affiliations:** † Centre for Sustainable & Circular Technologies, University of Bath, Bath BA27AY, U.K.; ‡ Department of Chemical Engineering, University of Bath, Bath BA27AY, U.K.; § Centre for Analysis and Synthesis, Department of Chemistry, Lund University, Lund 221 00, Sweden; ∥ Center for Nuclear Energy in Agriculture, University of Sao Paulo, Piracicaba 13400-970, Brazil

**Keywords:** cellulose acetate, nutrient, fertilizer, biopolymer, zinc salt, controlled release

## Abstract

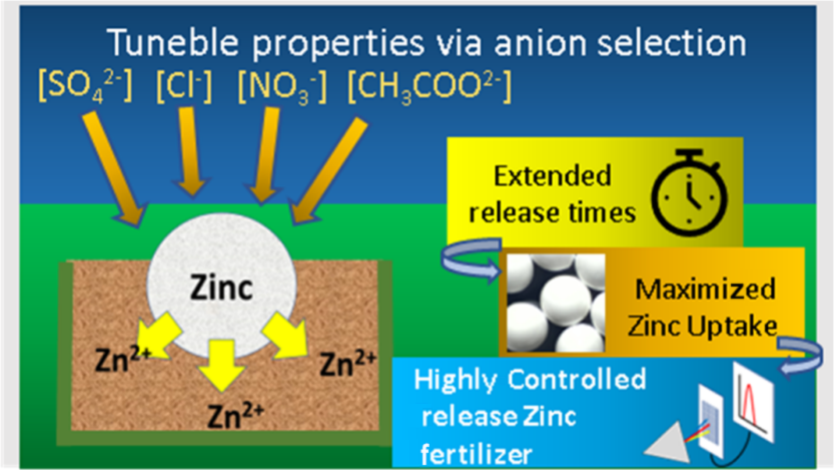

The controlled delivery of micronutrients to soil and plants is essential to increase agricultural yields. However, this is today achieved using fossil fuel-derived plastic carriers, posing environmental risks and contributing to global carbon emissions. In this work, a novel and efficient way to prepare biodegradable zinc-impregnated cellulose acetate beads for use as controlled release fertilizers is presented. Cellulose acetate solutions in DMSO were dropped into aqueous antisolvent solutions of different zinc salts. The droplets underwent phase inversion, forming solid cellulose acetate beads containing zinc, as a function of zinc salt type and concentration. Even higher values of zinc uptake (up to 15.5%) were obtained when zinc acetate was added to the cellulose acetate–DMSO solution, prior to dropping in aqueous zinc salt antisolvent solutions. The release profile in water of the beads prepared using the different solvents was linked to the properties of the counter-ions via the Hofmeister series. Studies in soil showed the potential for longer release times, up to 130 days for zinc sulfate beads. These results, together with the efficient bead production method, demonstrate the potential of zinc-impregnated cellulose acetate beads to replace the plastic-based controlled delivery products used today, contributing to the reduction of carbon emissions and potential environmental impacts due to the uptake of plastic in plants and animals.

## Abstract 2

Controlled uptake and release of zinc from cellulose acetate microbeads in both water and soil for precision agriculture.

## Introduction

The application and use of macro- and micronutrients in agriculture arises from the need to increase land productivity,^1^ improve plant health and defence against pathogens,^2^ and improve the intake of nutrients in human diets.^3^ While the primary fertilizers applied in agriculture are nitrogen, phosphorous, and potassium (NPK),^4^ micronutrients such as iron, manganese, boron, copper, and zinc are also necessary to promote healthy and progressive plant growth.^5^ Issues arise, however, from overapplication: Drainage water percolating through soil can lead to agricultural runoff, contributing to eutrophication of local water sources,^6^ leading to issues such as an alteration within the composition of photosynthetic organisms or harmful algal blooms.^7^ The use of water-soluble salts leads to leaching, low nutrient use efficiency,^8^ contamination of surface water and sediments,^9^ and accumulation of metals to toxic levels in local waterborne biomass.^10^ The manner in which these salts are applied will contribute to these issues: frequently, these will be applied alongside chelating agents such as EDTA which, in turn, have a detrimental effect on the environment by remobilizing metals, as well as an inherent resistance to biodegradation.^11^ Overuse of micronutrients during application can lead to their accumulation on foliar surfaces or within root structures, inducing cytotoxic responses within leaves,^12^ roots,^13^ or across whole plant systems.^14^

Controlling the release of nutrients can mitigate the adverse effects of nutrient application. Depending on the nutrient format—organic or inorganic compounds—this can be achieved in a number of ways, including via the use of polymeric^15^ and inorganic^16^ coatings and hydrophobic polymer matrices^17^ or otherwise altering the susceptibility of particles to biological or chemical degradation.^18^ However, the accumulation of microplastics in the oceans,^19^ in wildlife,^20^ and in agricultural crops^21^ is causing growing concern, with recent discoveries of microplastics in human placentae.^22^ As such, there is a renewed interest in the use of biodegradable polymer matrices as controlled release materials for the delivery of micronutrients. Several materials have been previously investigated, either as the sole component or as parts of compositions, including calcium alginate,^23^ chitosan,^24^ carboxymethyl cellulose,^25^ and zein.^26^ However, many of these biodegradable polymers have challenges blocking further adoption; for example, they are more expensive than oil-based materials.^27^ Cellulose is a potential alternative, being one of the world’s most abundant materials, accounting for 80% of biomass and is primarily derived from timber and cotton.^28^ The authors have recently demonstrated that cellulose can be used for the controlled delivery of micronutrients to plant roots from soil.^29^ However, cellulose is neither easily dissolved nor easily processed, requiring derivatization to undergo dissolution using common solvents or, in the absence of a derivatization regime, the use of expensive ionic liquids to dissolve standard cellulose.^30^ On the other hand, a number of cellulose derivatives—such as ethyl cellulose and cellulose acetate—are soluble in a wide range of solvents deemed “green/sustainable”, such as ethanol and DMSO.^31^ This could allow for the economic and sustainable deployment of cellulose-derived materials in the field of agriculture for the controlled-release of macro- and micronutrients.

The production of cellulose microbeads has been demonstrated via a range of techniques, including membrane emulsification,^32^ high-shear mixing,^33^ dropping (single channel extrusion), utilizing solvent/antisolvent precipitation by aqueous or organic solvents,^34^ and via electrospray.^35^ The choice of antisolvent will affect various properties of cellulose-based beads during the precipitation/regeneration process.^36^ However, there is very limited literature on the preparation of cellulose acetate beads. Previous studies include the use of solutions of cellulose acetate regenerated in an acidic antisolvent to form beads and to induce the production of gases from which internal pores are formed.^37^ Cellulose acetate beads have also been produced using high shear mixing to produce droplets, which can be stabilized and precipitated.^38^ However, beads produced in this manner have large voids in their center, which lead to their collapse into basin-shaped particles. Base-catalyzed deacetylation has been used to create cellulose acetate beads with large central voids, which have successfully been used to elute pharmaceuticals.^39^

In this work, a novel and efficient method to produce zinc-impregnated cellulose acetate beads was developed for the controlled release of zinc into soil. Zinc was chosen as a model micronutrient, as it is a key input in a variety of physiological processes in plants and yet is deficient in nearly half of the agricultural soils worldwide.^40^ The effects of solvent, antisolvent, and polymer concentrations on their size and internal structure were investigated to maximize the uptake of zinc and control its release. Porous cellulose acetate beads were produced by the addition of various zinc salts to the antisolvent during regeneration. The uptake of zinc into the beads was measured, with a comparative study on their release in aqueous and soil-based environments showing the possibility of controlled release over a long period of time.

## Methods and Materials

### Materials

Cellulose acetate (Mn 30,000) (9004-35-7) and zinc nitrate (228737) were obtained from Sigma-Aldrich. Zinc chloride (98%+), zinc sulfate (99%), and zinc acetate (98%) were obtained from Fisher Scientific. Sodium chloride (S9888), zinc carbonate (96466), and DMSO (technical grade) were obtained from Honeywell Ltd. Ultrapure DI water was produced using Veolia PURELAB Chorus (resistivity 18.2 MΩ).

### Methods

#### Production of Beads via the Dropping Method

Pure cellulose acetate beads were made as follows: solutions of cellulose acetate were prepared in volumes of 50 mL using DMSO, with dissolution initiated with sonication (10 min, 40 °C, 37 kHz) followed by roller mixing (24 h, room temperature). Droplets were produced via a needle dropping method using needles with internal diameter 18–30 G (0.838–0.159 mm, stainless steel, Bauer). Using a syringe pump, the cellulose acetate solutions were extruded through a needle into a pool of antisolvent (DI water) below. The needle/antisolvent distance was 60 mm to ensure production of spherical beads. This optimal needle height was determined experimentally using eq S1 to determine the maximum needle height and droplet velocity and ensure that any particles produced were spherical. Needle size and solution viscosity also contribute to obtaining spherical beads, as well as determining the size of the droplet produced.^41^ Precipitated beads of cellulose acetate were left to settle for 24 h in the antisolvent solution, before washing with fresh antisolvent twice more for 12 h each, before retrieval by filtration. Beads were dried at 50 °C for 48 h within a drying cabinet and were weighed periodically for the final 24 h until no further mass loss was observed. Two solution concentrations of cellulose acetate (10 and 15% wt) were assessed to determine their suitability as solutions for spherical bead production.

Impregnation of zinc into cellulose acetate beads was achieved via two different methods: (i) precipitation of cellulose acetate droplets in zinc-containing aqueous antisolvents and (ii) addition of zinc salts into the cellulose acetate solution followed by precipitation in zinc-containing antisolvents. Using the dropping method for cellulose acetate (15% wt in DMSO), the droplets were regenerated in solutions of zinc salts (1–12% wt zinc), followed by further rinsing in antisolvent (500 mL per 3 g of beads, twice) to remove DMSO. The second method used the same antisolvent method to impregnate beads with zinc, with the addition of zinc acetate within the cellulose acetate solution (prepared to 2% wt zinc, with 15% wt cellulose acetate in DMSO). The beads were then dried as per the previous method. All soaking was conducted in the appropriate zinc salt solution to achieve phase inversion and zinc uptake.

### Characterization Methods

The viscosity of the polymer solutions was measured using a Discovery Hybrid Rheometer (HR-3, TA Instruments) using a sand-blasted 40 mm 1° cone at a gap of 50 μm. Analysis was conducted by flow sweeps at shear rates between 0.01 and 100 s^–1^, at 25 °C (±0.1 °C). Cross-sectional imagery of the beads was obtained using X-ray computed tomography, using a Nikon XT H 225 ST CT scanner using a Tungsten target and PerkinElmer 1620 16 bit, 2000 × 2000-pixel detector. Scans were processed using Avizo Fire (v8.0). Scanning electron micrographs were obtained using a JEOL 648 OLV scanning electron microscope. Sample preparation involved freeze-drying and rupture to obtain access to inner portions of the beads, followed by sputter coating prior to imaging (gold, Edwards sputter coater). Mechanical analysis of dried beads was performed using an Instron 3369 at a rate of 0.1 mm min^–1^, using a 100 N load cell. Bead sizes were measured using digital callipers (RS Pro ±0.01 mm) with sample averages obtained for *n* = 10–15. IR spectra were obtained using attenuated total reflectance FTIR (Bruker Alpha II).

#### Determination of Beads’ Zn Content, Relative Rate of Release, and Release Profile

Dried beads produced using various zinc-containing antisolvents were immersed in water, under constant stirring. Conductivity measurements were taken every 3 s using the Accumet 1330 software, allowing for end-point determination of zinc release against the pre-prepared calibration curves. The limit of detection was determined to be 0.001% wt Zn, with the limit of quantification determined for all zinc salts set at 0.003% wt. The quantity of water (1 L) against the quantity of beads used in each test (∼0.3 g) ensured that any zinc remaining in the beads at the end point was, therefore, negligible.

#### Zinc Release from Beads in a Soil Matrix

**Figure 1:**
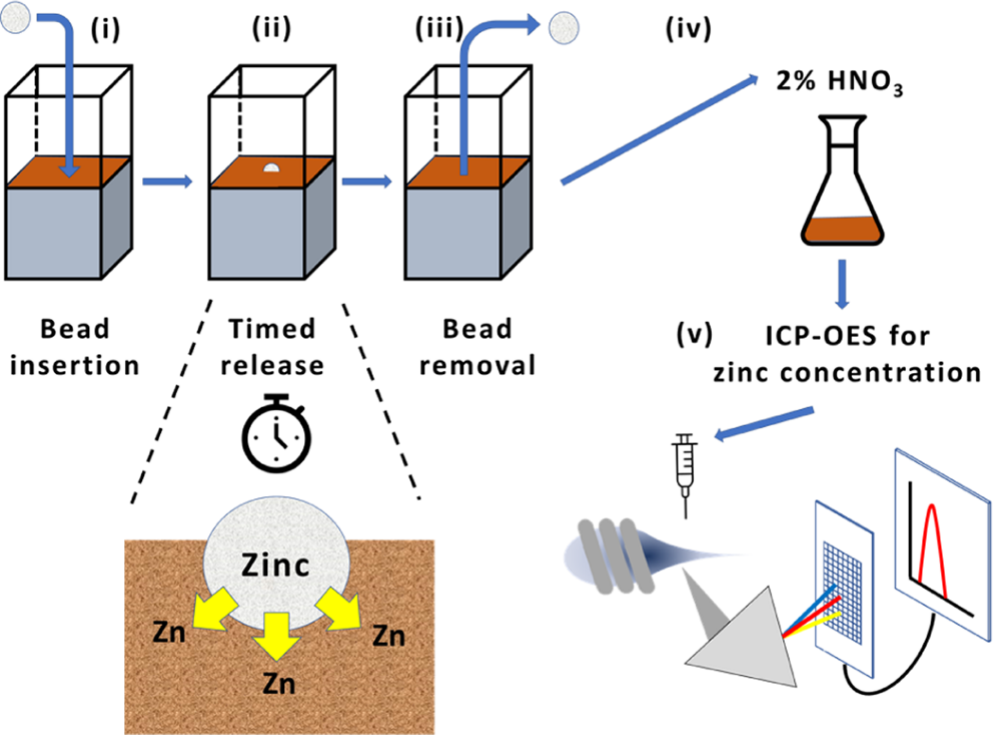
Flow diagram of the process to determine the quantity of zinc released from beads into surrounding soil over time: (i) beads are inserted into soil for a specified period of time; (ii) zinc enters the soil; (iii) beads are then removed at a predetermined time; (iv) soil is then digested according to ISO 17586; and (v) extract is analyzed using ICP-OES.

The soil for zinc-release tests was obtained from the Salisbury Plain, UK, at a location that is not subjected to dispersion of fertilizer. Soil pH was determined by preparing soil samples in 0.01 M calcium chloride solution at a ratio of 1:5 (by volume), using a glass electrode as per ISO 10390:2021. This utilizes solutions of barium sulfate, magnesium sulfate, and sulfuric acid to determine the ionic strength of the soil, combined with pH measurements of extracts. The samples were prepared in triplicate. The beads were dried at 50 °C for 48 h within a drying cabinet and were weighed periodically for the final 24 h until no further mass loss was observed. The release of zinc from the beads into a soil matrix was performed using soil samples maintained at 25% hydration. Soil cuvettes were 3D-printed using a Stratasys F 270 FDM unit using PLA filament (Figure 1i–iii). These were prepared with voids at the bottom of the container and a 2 mm sponge layer along the bottom to allow for adjustment of the soil water content and to reduce drainage. The cuvettes were then filled with soil and placed in a humidity-controlled test chamber (TAS Ltd.) and programmed to retain a 25% relative humidity using a Rotronic controller. The beads were placed in soil samples for specific time periods, after which they were removed and the soil digested as per ISO standard 17586, utilizing nitric acid (2%) and agitation (2 h, end over end). Once digested, the samples were centrifuged (1000 rpm), and the leachate was filtered (0.45 μm PTFE membrane filter). This was then analyzed for zinc content by ICP-OES (JY Horiba Ultima 2c ICPOES) using procedural blanks (*n* = 3), soil background blank (*n* = 5), and spiked soil samples (*n* = 6) as quality assurance for the analysis (Figure 1iv,v).

## Results and Discussion

### Characterization of Cellulose Acetate Beads

**Figure 2:**
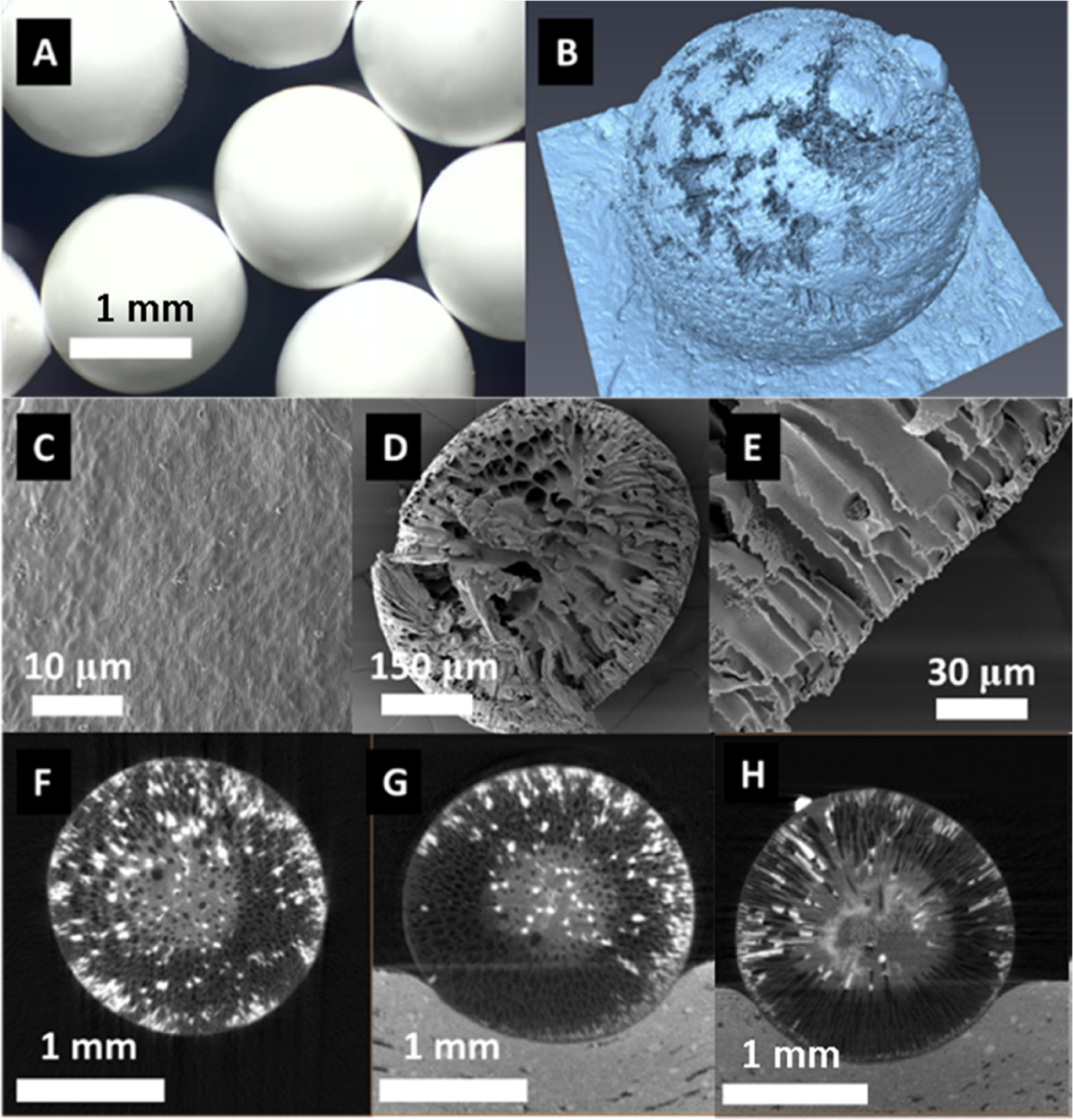
Cellulose acetate beads produced using 15% wt cellulose acetate in DMSO, regenerated in deionized water: (A) optical micrographs, (B) composite 3D image taken using X-ray computed tomography, (C–E) SEM micrographs, and (F–H) X-ray computed tomography of the beads along the *X*, *Y,* and *Z* axis, highlighting the uniformity existing within the beads produced by this method.

Using 15% wt cellulose acetate solution in DMSO, beads were produced via the dropping method and characterized. Optical imagery shows spherical white beads with a smooth surface (Figure 2A). Investigation of the outer bead structure using X-ray computed tomography identified the presence of variances in the depth of the outer shell of the beads (Figure 2B). Scanning electron microscopy of the beads’ surface and cross-section shows that the outer surfaces of the beads are smooth (Figure 2C) with internal macro- and microporous structures (Figure 2D,E). The bulk of the porous structures emanate from a central mass, extending toward the surface where narrower voids exist below the surface of the beads. Identified centrally within the bead was a central mass of polymer, with finger-like porosity emanating from this central core (Figure 2F–H). It is envisaged that this pore structure is due to the rate of mass transport, which occurs during droplet precipitation, where an initial outer shell is formed, reducing the speed at which the solvent–antisolvent exchange occurs, giving rise to the denser, solid core.

### Bead Sphericity

**Figure 3:**
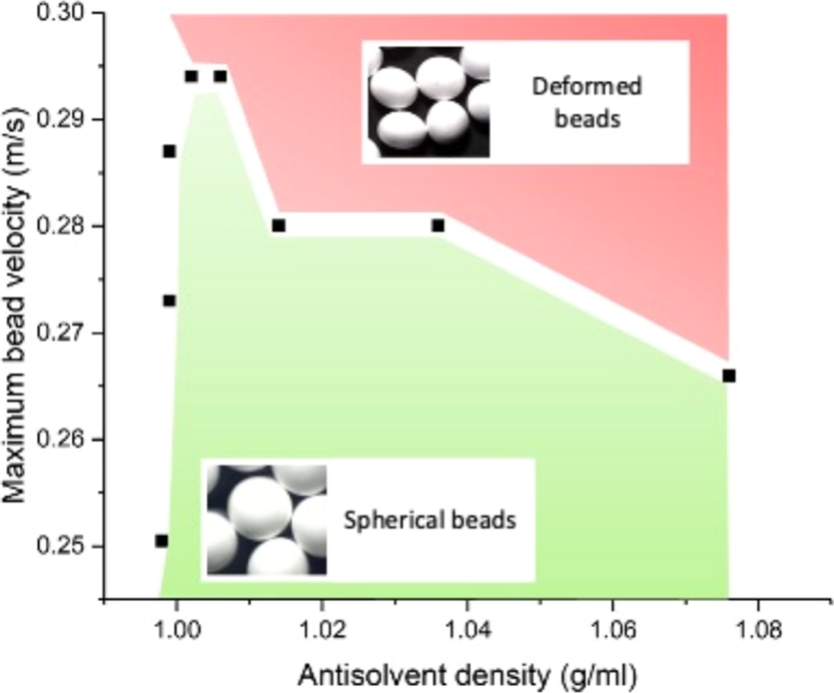
Bead sphericity as a function of antisolvent density and velocity of droplets entering antisolvent solution, providing the operational parameters for spherical droplet production by the needle dropping method.

The effect of the operational parameters on the sphericity of the beads obtained by the dropping method was examined by assessing the effects of both the antisolvent density and the needle-to-antisolvent distance (Figure 3). Bead sphericity is important for slow-release fertilizers as it contributes to uniform release over time and ease of distribution via mechanical means.^18^ It was observed that antisolvent density had a profound effect on the ability to produce spherical beads. An increase in the antisolvent density above 1.01 g/mL required a lower distance between the needle and the antisolvent to ensure bead sphericity. These distances were used to determine the droplet velocity at impact, which was envisaged to exert an influence on the sphericity of the droplets once they enter the antisolvent, due to the increasing energy of the impact at higher speeds. Higher needle-to-antisolvent distance deformations occurred on impact, resulting in disc- or pancake-like beads (Figure 3).

#### Zinc Uptake and Release

In a previous work by the authors, cellulose beads were first formed by precipitation in water, dried, and then immersed in a zinc solution to impregnate them with the metal.^29^ Here, impregnation has been made more efficient by precipitating the cellulose acetate droplets directly into an aqueous antisolvent containing a zinc salt. Initial experimentation using zinc chloride highlighted that zinc uptake in the beads increased with increasing concentration of zinc within the antisolvent. Further, zinc salts (carbonate, sulfate, acetate, and nitrate) were examined to ascertain if this trend was applicable to zinc species with different counter-anions. These were chosen to ensure that they met environmental regulations and were generally regarded as safe (GRAS) by the Food and Drug Administration,^42^ with no significant concerns for human exposure, environmental fate, ecological effects, or effects on animals grazing in areas with zinc salts applied.^43^ Zinc products have been approved for use as pesticides, herbicides, and fungicides by US state agencies.^44^ This is primarily due to the binding potential of these zinc compounds in soil, which is dependent on soil texture, pH, organic carbon content, and soil cation exchange capacity.^45^ Zinc salts (notably zinc chloride, despite its potential to cause damage to aquatic life) are added to irrigation water to increase the micronutrient content of soils.^46^

**Figure 4:**
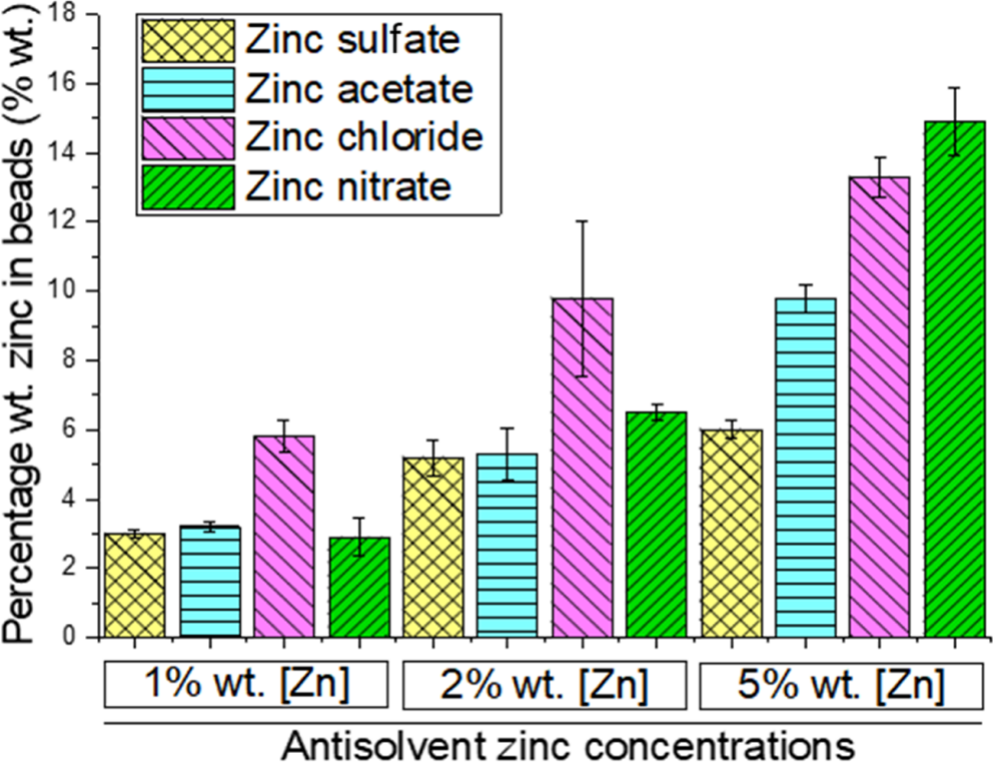
Zinc uptake in cellulose beads (produced using 15% wt cellulose acetate in DMSO) across four different zinc salts measured using conductivity. Antisolvent concentrations were normalized to zinc concentration.

The capacity for cellulose acetate beads to uptake zinc was initially tested across the five zinc salts at various concentrations of 1, 2, and 5% wt of zinc [Zn], with significant variations observed across the different zinc species (Figure 4).

Of the five separate antisolvents tested, zinc carbonate was rejected early on as this formed a dispersion in water rather than a solution at concentrations of 1–5% wt [Zn], and so was discarded from further assessment. The remaining zinc salts—sulfate, nitrate, chloride, and acetate—were assessed to determine the effect that the counter-anion had on zinc uptake during regeneration. At 5% wt [Zn], the uptake in beads ranged from concentrations of 5.0 to 14.9% wt [Zn]. The highest concentration recorded was produced using zinc nitrate; however, upon drying, these beads become brittle, deformed, and discolored (Figure S1). This made the nitrate salt be discounted as a compound of interest. Zinc chloride was tested because of its high solubility in water; however, upon testing zinc chloride concentrations above 5% wt [Zn], the beads were found to be prone to hydrolysis during drying. This resulted in beads decomposing during the drying phase (Figure S2). Aqueous zinc chloride is a known solvent for both cellulose and cellulose acetate and acts as a mediator for acid hydrolysis, which reduces the effectiveness of this compound at higher concentrations despite promising initial results.^47,48^ Zinc acetate was found to have high solubility in water; however, solutions of zinc acetate rapidly become too dense to support the production of spherical beads: the impact of the droplets on the surface of the zinc acetate antisolvent solutions at concentrations greater than 5% wt [Zn] resulted in oblong spheroid shapes rather than spherical beads (Figure 3 top). As discussed previously, to counteract increasingly denser antisolvents, the needle–antisolvent distance must be reduced; however, this has the effect of approaching a minimum threshold distance, which prevented droplets from resolving into spheres prior to the impact on the surface of the antisolvent. For this reason, zinc acetate antisolvent concentrations were limited to 5% wt [Zn]. It was found that zinc sulfate solutions of 12% wt [Zn] could support bead formation; however, this method only produced zinc-uptake concentrations of 12.5% wt [Zn], lower than that achieved by 5% wt [Zn] antisolvents when zinc nitrate or zinc chloride was used (Table 1).

**Table 1:** Thermochemical Properties of Zinc Counter-Anions (Δ*H*_hyd_ is Molar Gibbs Energy of Hydration of Ions,^49^ and *P* is the Radial Charge Density;^50^ Maximum Zinc Uptake in Beads at 5% wt [Zn] in the Antisolvent; [Max] is the Maximum Possible Zinc Concentration in the Antisolvent that Allowed the Production of Beads)

	zinc salts		beads	antisolvent
counter-anion	Δ*H*_hyd_(kJ mol^–1^)	*P* (C m^–1^)	zinc uptake in beads (% wt [Zn])	[max] (% wt Zn)
nitrate ion	–300	–5.78	14.9	5%
chloride ion	–340	–6.25	13	5%
acetate ion	–365	–7.12	9.8	5%
sulfate ion	–1080	–8.19	6.0	12%

**Figure 5:**
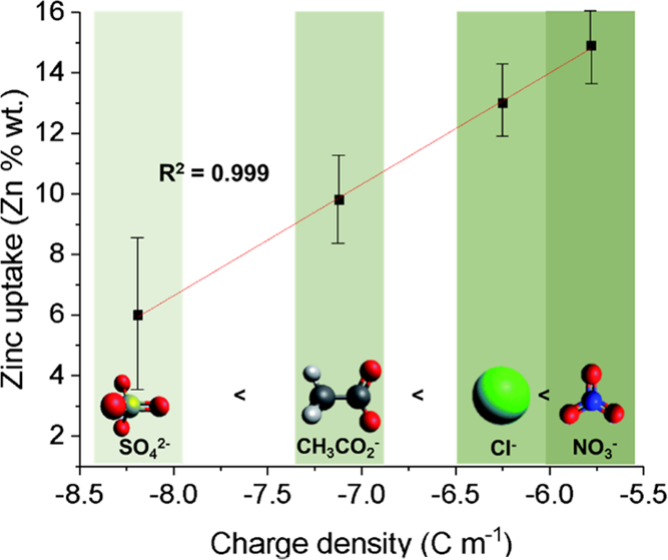
Relationship of radial charge density (coulombs per meter) of zinc counter-anions with zinc uptake (measured in % wt zinc within the beads) in cellulose acetate beads.

The order of increased uptake of these anions was found to follow the lyotropic and Hofmeister series,^51^ primarily dictated by the constituent atoms of the ions.^52^ The mechanism of uptake is envisaged to occur due to the polymer–salt interactions that occur while the polymer undergoes phase inversion from solution. These polymers provide areas for aggregation of the zinc salts at the phase boundary of the cellulose acetate and zinc solutions. This has been seen in DFT studies on cellulose acetate–Zn^2+^ interactions, where the carbonyl groups of the acetate provide an electronegative attraction to the positively charged zinc ions. It was found that Zn^2+^ forms 4-coordination bonds with the surrounding carbonyl groups.^53^ As the precipitation of the cellulose acetate droplets happens rapidly while the exchange of the solvent/antisolvent occurs, this provides nucleation sites for the zinc salts to aggregate. The droplets undergo phase inversion into beads over the 48 h of soaking. Upon drying, these zinc salts crystallize at these likely sites of interaction along the cellulose acetate molecular chain. A strong correlation was found where zinc uptake in the beads increased with increasing magnitude of the radial charge density *P* of the anions (Table 1 and Figure 5). The trend broadly followed the Hofmeister series (SO_4_^2–^ < C_2_H_3_O_2_^–^ < Cl^–^ < NO_3_^–^).^51^ The increase in zinc uptake as the anion charge density decreases in magnitude suggests that an increased charge could inhibit the diffusion of the anion into the particles at the point of phase inversion of the droplet of cellulose acetate solution, or during later stages of soaking. The increasing energy required to remove anions of higher magnitude from hydration could work in parallel with the preference for salts to form interactions with different moieties, as observed for anion uptake in other systems.^51^ Overall, the direct impact of the counter anion appears to determine the quantity of zinc that is taken up by the beads during regeneration which, in turn, affects the total zinc available for release, and the rate at which it egresses from the beads.

To further increase the zinc uptake, solutions of cellulose acetate (15% wt) with zinc acetate (1 and 2% wt) in DMSO were produced (16 and 17% wt total solids overall), with droplets of these precipitated in aqueous ZnOAc_2_ antisolvent solution (5% wt Zn). These produced beads with zinc content of 11.7 and 15.5%, respectively, higher than the values obtained for the ZnOAc_2_ antisolvent alone (cfr. Table 1).

#### Release Profiles for Zn Salts

**Figure 6:**
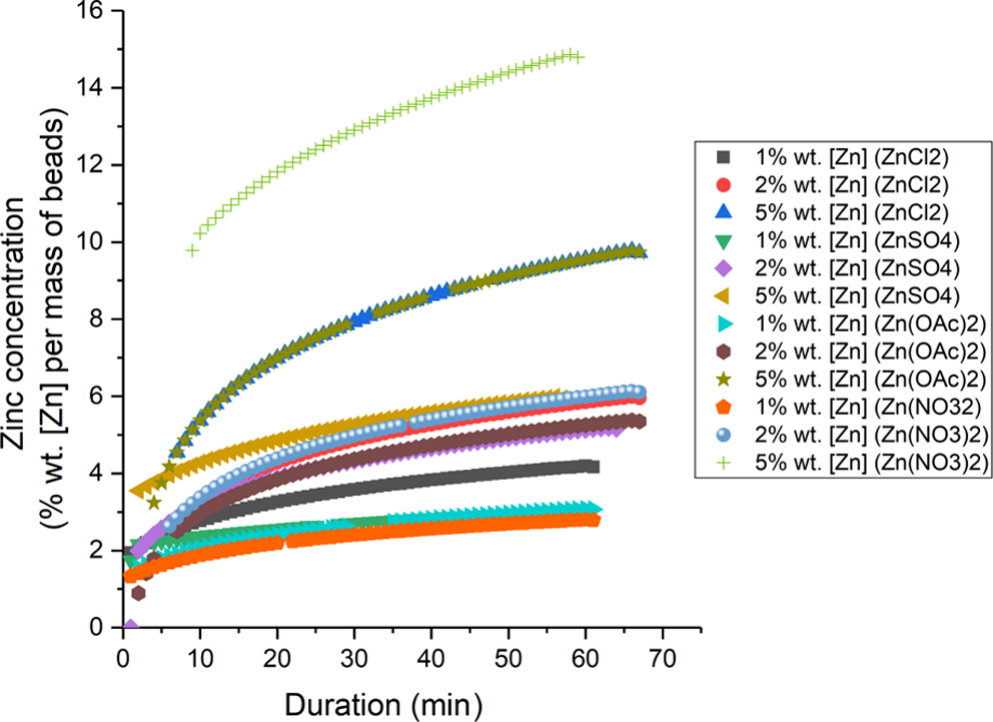
Release profiles for zinc chloride, zinc sulfate, zinc acetate, and zinc nitrate at 1, 2, and 5% wt [Zn].

The release of zinc from the beads in distilled water shows that the rate of release is dependent on both the exact zinc compound used, and the concentration of the zinc within the beads (Figure 6). No significant difference in diameter could be ascertained across the beads produced using the various zinc salts (Figure S3), which allowed discounting bead diameter as a potential source of variation for total zinc-uptake during precipitation, nor for the variation in zinc release in water (Table S1). Using the data in Table S1, comparing the retention times of beads precipitated in 1 and 5% wt zinc salts shows that the beads with a higher concentration tended to elute their zinc over a slightly longer time period (between 10 and 12% longer on average between 1 and 5% wt). Release profiles for zinc salts across all beads in water showed a range of 47 to 67 min release time (Table S1).

The steep release curves observed in the first few minutes of the release experiments followed by slower rates could indicate the possibility of “burst release” kinetics as soon as the beads are immersed in the water bath, in analogy to what is observed in drug release.^54^ This is a phenomenon that is witnessed when charged ions within delivery substrates are exposed to aqueous surroundings, causing a rapid migration from the substrate.^55^ The comparison between 1% wt zinc sulfate and zinc acetate shows that although both antisolvents can impregnate similar amounts of zinc within the beads (Figure 4) and have similar release profiles (Figure 6), the sulfate beads eluted over a shorter time period (Table S1). This difference in retention time suggests that the associated anions impart a physical or chemical modification, which inhibits, or induces, the release of zinc. This is perhaps a result of the difference in charge densities between the SO_4_^2–^ and OAc^–^ anions (cfr. Table 1), or due to the difference in mobility between the monovalent acetate and bivalent sulfate anion.^56^ The release of the Zn^2+^ cation itself will depend on the nature of the sorption, which causes the cellulose acetate to retain the zinc: density functional theory studies have shown that within aqueous environments, the interactions of Zn^2+^ and cellulose acetate occur in oxygen atoms throughout the polymer and that the formation of complexes between cellulose acetate and Zn^2+^ ions occurs spontaneously. These studies highlighted that this interaction is partially covalent and an additional H-bond appears, suggesting the formation of zinc–cellulose complexes that have been reported elsewhere.^53^

### Soil Release Tests

**Figure 7:**
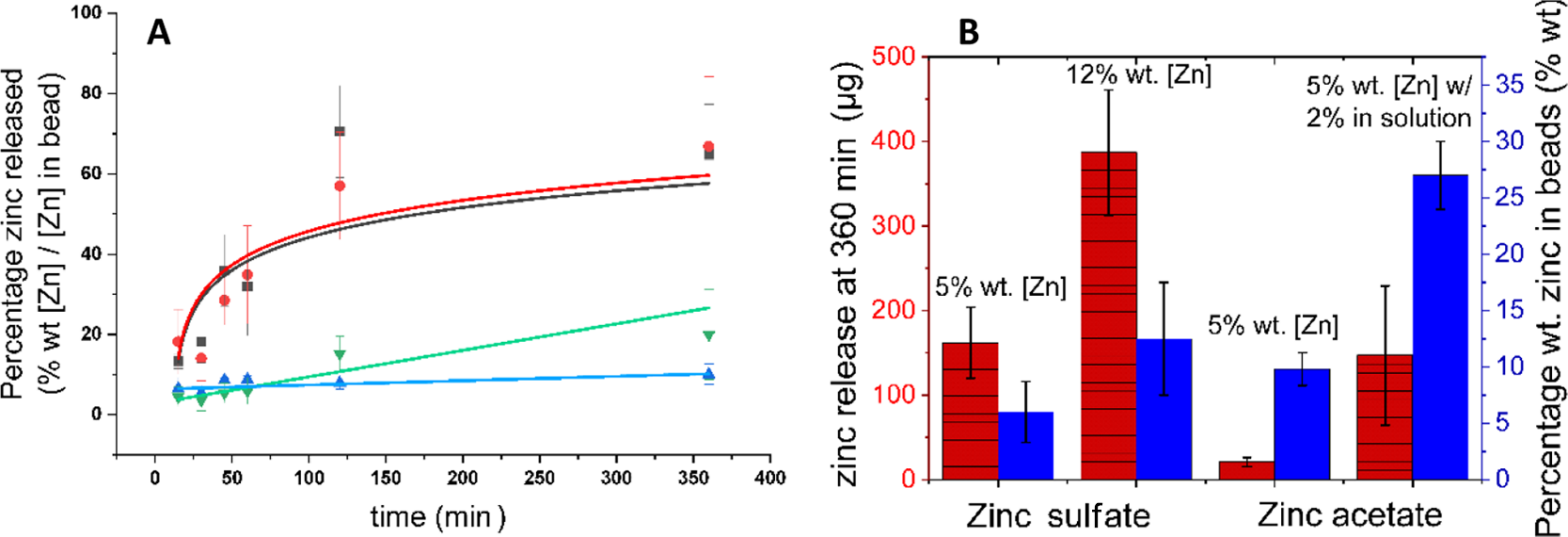
(A) Total release of zinc from beads produced by four different methods, with beads produced in (black, ■) 12% wt [Zn] zinc sulfate, (red, ●) 5% wt [Zn] zinc sulfate, (blue, ▲) 5% wt [Zn] zinc acetate, and (green, ▼) 5% wt [Zn] zinc acetate (w/2% wt [Zn] zinc acetate in polymer solution) and (B) zinc release as total mass released at 360 min (red column) and as the initial loading (blue column) for all four bead samples used in soil-matrix release testing. Error bars represent standard deviation.

The release of zinc from cellulose acetate beads in soil was examined by placing the beads in a soil matrix for specific durations, with roughly 60% of the bead submerged within the soil (Figure 1), and measuring the increase in the zinc content present in the soil over time (Figure 7b). This was conducted in triplicate for durations of 15, 30, 45, 60, 120, and 360 min. Four different types of beads were tested: beads prepared using 15% wt cellulose acetate in DMSO solution, utilizing (A) 5% wt [Zn] and (B) 12% wt [Zn] zinc sulfate antisolvents; (C) 5% wt [Zn] zinc acetate antisolvent; and (D) beads prepared using 15% wt cellulose acetate solution plus a further 2% wt [Zn] zinc acetate, utilizing 5% wt [Zn] zinc acetate antisolvent. Their relative release in soil was calculated by reversionary assessment: a maximum potential concentration of zinc per bead (or unit weight of beads) was determined initially using the concentration of the zinc in the beads released into water (as per Figure 6). The subsequent determination of zinc released into soil at timed intervals via soil digestion could then be weighed against this “maximum potential zinc” concentration to ascertain what percentage of the total zinc was released at any given time. This maximum value of zinc in the beads was then used to extrapolate release rates and estimate the timescales for total zinc release.

The overall release of zinc from the beads produced using the sulfate and acetate beads shows that there were significant differences in their respective rates of release and the total zinc released over 360 min (Figure 7A). Taking beads containing zinc sulfate, it was found that these released approximately 65% of their total zinc content at 360 min. The acetate beads were found to retain more of their zinc: at the 360 min mark, there was a release of only 10% (5% wt [Zn] Zn(OAc)_2_) and 20% (5% wt [Zn] Zn(OAc)_2_ w/2% [Zn] in polymer solution) for these beads (Figure 7A). Fitting of these curves to determine their rates found that the release was linear for both sets of acetate-containing beads, whereas the sulfate curves show a logarithmic rate of release (Figure 7A).

Using fitted curves for the zinc-release data in Figure 7A, it is estimated that the zinc sulfate samples will release their total zinc over a time span of 47 days for the beads produced in 12% wt [Zn] zinc sulfate, while a shorter duration of 32 days was estimated for beads produced in 5% wt [Zn] zinc sulfate (Table 2). While it may be expected that beads with a higher zinc content should possess longer elution times, the opposite was observed. This could be attributed to the beads with highest zinc concentration (those produced using 12% wt [Zn] zinc sulfate solution) having a higher concentration gradient between the beads and the surrounding soil, leading to quicker initial release over the 6 h of measurement. This could also be in part due to the osmotic difference pulling water toward the beads, facilitating higher rates of transport from the bead during the early stages. In comparison, the beads produced in zinc acetate showed different release profiles: the beads produced using 5% wt [Zn] (Zn(OAc)_2_ w/2% wt [Zn] in polymer solution had a predicted release time of ∼2.5 days until full release of all zinc within the beads, while those produced using 5% wt [Zn] (Zn(OAc)_2_) have a ∼16 days time span before full release of all zinc (Table 2).

**Table 2:** Fitted Curves with r-squared Values, Residual Sum of Squares(*), Reduced Sum of Squares(**), and Calculated 100% Release Times for Four Zinc Bead Formats

antisolvent used	fitted eqn	*R*^2^	estimated time until 100% release
12% wt [Zn] zinc sulfate	*y* = 9.8* ln(*x* – 11.22)	0.80	1,125 h (∼47 days)
5% wt [Zn] zinc sulfate	*y* = 10.18* ln(*x* – 11.04)	0.78	18,500 h (∼32 days)
5% wt Zn(OAc)_2_	*Y* = 6.34 + (0.01 *x)	0.75	390 h (∼16 days)
5% wt Zn(OAc)_2_ w/2% [Zn] in polymer solution	*y* = 2.8 + (0.07 *x)	0.75	57 h (∼2.5 days)

The release from the 12 and 5% wt [Zn] zinc sulfate samples (Figure 7A) has a similar profile to what is observed in Figure 6, suggesting that “burst release” might have occurred in this situation as well, with ions at the surface transported to the surrounding soil at a higher rate than the zinc contained deeper within the beads. A simple interpretation of the results in Figure 7A,B is further complicated by other factors: although the decomposition of cellulose acetate by biological/bacterial means under aerobic conditions is well-known,^57^ this degradation is dependent upon numerous factors, including the degree of substitution, the size and shape of the material, the biological species present, the immediate chemical environment. and temperature.^58^ As such, it could be expected that the rate of release could increase as decomposition occurs; however, this could not be experimentally determined. The difference in release profiles for both acetate and sulfate could be also influenced by soil pH, which was determined experimentally to be pH 6.8. It is known that Zn^2+^ ions entering soil matrices may bind to organic matter (e.g., humic and fulvic acids and other materials that act as chelating agents) and onto hydroxides present in the soil, existing in equilibrium between these solids and the water present in the interstitial voids within the soil.^59^ Soil–water partition coefficients for zinc species show that zinc ion solubility increases by a factor of 5 with every pH unit decrease. These data imply that there is an advantage for zinc sulfate with regard to diffusion from cellulose acetate beads to the immediate aqueous environment due to its pKa value, which in turn suggests that sulfate-based zinc compounds will undergo diffusion and transfer from within bead to the immediate soil–water matrix at a rate that is greater than that for the zinc acetate beads. As such, higher pKa values could potentially ensure a longer release time due to the lower dissociation constant for the said salts. It could also be posited that the overall solubilities of the various zinc compounds in water affect these results: the solubility in water for the acetate dihydrate variant (43 g/100 mL) is lower than that for the sulfate heptahydrate variant (54 g/100 mL).^60,61^ This increase in solubility could explain the increased speed at which the zinc sulfate entered the surrounding moist soil, aided by the osmotic pressure.

A direct comparison with other controlled release fertilizers is made complex not only from the wide range of supports and materials but also from the large number of factors that can affect release, including the rates of biological and chemical degradation of the beads, the form of zinc salt present, and the presence of water in the form of humidity. For example, zinc compounds such as zinc chloride and nitrate are known to enhance the chemical breakdown of cellulose and its derivates,^47^ showing that the presence of zinc initiates an acid hydrolysis reaction at room temperature, degrading the glycosidic bond and producing glucose.^47^ In such a case, it could be expected that the hostile conditions created by the presence of zinc chloride (or other zinc compounds) within the bead could expedite the physical deterioration of the bead chemically or encourage biological degradation through the production of sugars. Additionally, oligomers produced during the bead decomposition might enhance the soil cation exchange capacity, which is a highly desirable feature capable of improving the retention of other nutrients. Furthermore, such oligomers, likely negatively charged, may also prevent phosphorus strong adsorption on iron and aluminum oxides. These two hypotheses shall be tested in further studies. Other factors affecting Zn^2+^ release from the cellulose substrate include local salinity and pH, as other lignocellulosic materials have shown to have preference for retention of other bivalent metal ions under specific salinity and alkaline conditions.^62^

To date, this is the first example within the literature of a controlled release fertilizer produced using cellulose acetate as the sole matrix component. Comparing this material against other composite materials as controlled release fertilizers is favorable toward the cellulose acetate bead method: zinc sulfate encapsulated within manganese hollow-core shells was released within ∼33 days,^63^ a shorter timespan than that reported in this study. The release of zinc sulfate from beads produced using microcrystalline cellulose occurred within ∼400 min within both loamy and sandy soils.^29^ Other formats involve coating urea with Zn-fortified nano-bentonite and ZnO nanoparticles using various binders which could release zinc, but with full zinc release occurring at around 30 days.^64^

### Compression Testing

Compression testing carried out on the cellulose acetate beads of diameters ranging from 1.75 to 1.93 mm required ∼10 N to achieve approximately 30% compression (Figure S4), with no fracturing occurring under loading. Previous works on the mechanical strength of cellulose microbeads found that these also exhibited no fracture at compression, with 59 N required to achieve compression at 30%.^32^

## Conclusions

In this study, a novel method with potential for scale-up to produce zinc-impregnated cellulose acetate beads was developed using zinc-containing antisolvents to induce uptake during regeneration. Increased levels of encapsulated zinc compounds were achieved by utilizing zinc salts that possessed counter-anions with lower magnitudes of radial charge densities, by using different concentrations of cellulose acetate and by impregnating cellulose acetate solutions with zinc acetate prior to precipitating the droplets of this solution in zinc-containing antisolvents. Both the quantity of zinc within these beads and their release time in aqueous environments was found to depend highly on the form of the zinc salts present, with this release monitored by conductivity measurements to track the concentration of the zinc released. The level of zinc impregnation achieved surpass that of cellulose beads reported in the literature, with release times from the beads exceeding recent zinc delivery methods. Release tests in soil showed slow delivery over time for both zinc sulfate and zinc acetate beads, with the potential to deliver zinc ions to soil over timescales exceeding 45 days. These results show that the controlled release of micronutrients can be achieved using a biodegradable carrier, potentially eliminating the need to use the fossil-fuel-derived plastics currently used in agriculture.

## Supporting Information Available

Release time for zinc from beads based on antisolvent used, beads produced with zinc nitrate, decomposed cellulose acetate beads produced in zinc chloride antisolvent, average diameters of cellulose acetate beads produced using 15% wt. cellulose acetate solution in DMSO, and compression testing of two cellulose acetate beads (PDF)
