# Supplementary material for: Cellulose Acetate Microbeads for Controlled Delivery of Essential Micronutrients
Source: ACS Sustain Chem Eng. 2023 Mar 14;11(12):4749–58. doi: 10.1021/acssuschemeng.2c07269 (PMC10052346; doi:10.1021/acssuschemeng.2c07269)
Supplement: Supplementary file 1 — sc2c07269_si_001.pdf [file sc2c07269_si_001.pdf]

Supplementary Information
Cellulose Acetate Microbeads for Controlled Delivery of Essential Micronutrients

Ciarán Callaghan,[‡§] **Davide Califano,**[‡] **Marcos Henrique Feresin Gomes,**[ǁ] **Hudson Wallace Pereira de Carvalho,**[ǁ] **Karen J Edler,**[‡ ˧] and **Davide Mattia.**[‡§*]

3 pages, 4 figures, 1 table

Methods:

Determining effect of antisolvent density on sphericity of beads:

This method set out to ascertain the optimum bead height for producing spherical cellulose acetate beads using the dropping method. Using eight antisolvents of various densities (using sodium chloride solutions) the distance between needle tip and surface of the antisolvent was varied until perfectly spherical droplets were produced. These dropping heights were initiated at 60 mm above the surface of the antisolvent and dropped down in 5 mm increments until spherical droplets were retrieved from the antisolvent solution. Once spherical beads were identified, the height was increased by 2 mm to find the maximum height with which spherical beads could be produced. Once the distance was established, the velocity of the beads at impact was determined using the following equation:

$$v = \sqrt{2gd} \qquad \text{(eqn.S1)}$$

Where *v* is velocity (m/s), *g* is the gravitational constant, and *d* is antisolvent-needle distance (m).

Results:

Ensuring that droplets entering the antisolvent retain their spherical nature requires that a) these droplets are spherical at the point of reaching the surface of the antisolvent, b) that the velocity of the droplets is not great enough to deform them on impact with the antisolvent or c) the density of the antisolvent is not too great that the antisolvent-needle distance needs to be reduced to the point where it matches the minimum permissible distance. Experimental work using antisolvents of density 0.998 – 1.075 g/ml found that as antisolvent density surpasses ~1 g/ml, the antisolvent-needle distance (and therefore the maximum permissible velocity of bead at impact) must be reduced in order. Using this method, the use of 10% wt. cellulose acetate in DMSO was found to be sensitive to dropping distance, and a higher concentration cellulose solution was used in the rest of the work (15% wt. cellulose acetate).

Table S 1. Release times for zinc from beads by their antisolvent used.

Antisolvent used	Release time (m)	Antisolvent used	Release time (m)
1% wt. [Zn] ($ZnCl_2$)	61	1% wt. [Zn] ($Zn(OAc)_2$)	60
2% wt. [Zn] ($ZnCl_2$)	67	2% wt. [Zn] ($Zn(OAc)_2$)	66
5% wt. [Zn] ($ZnCl_2$)	66	5% wt. [Zn] ($Zn(OAc)_2$)	67
1% wt. [Zn] ($ZnSO_4$)	47	1% wt. [Zn] ($Zn(NO_3)_2$)	60
2% wt. [Zn] ($ZnSO_4$)	64	2% wt. [Zn] ($Zn(NO_3)_2$)	66
5% wt. [Zn] ($ZnSO_4$)	55	5% wt. [Zn] ($Zn(NO_3)_2$)	59

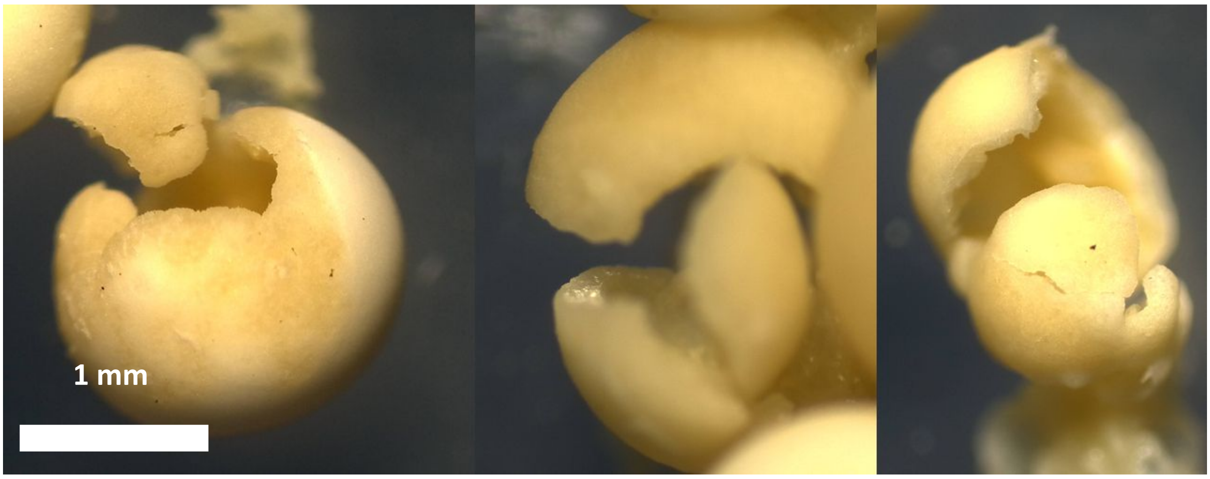

Figure S1. Images of beads produced with zinc nitrate, which have cracked during drying, exposing their hollow inner shall. Imaging taken on a Evos 1.01 Microscope system.

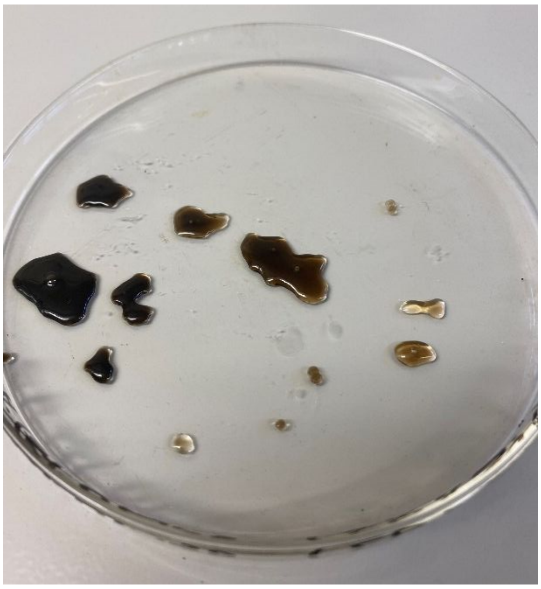

Figure S2. Decomposed cellulose acetate beads which were produced in zinc chloride antisolvent

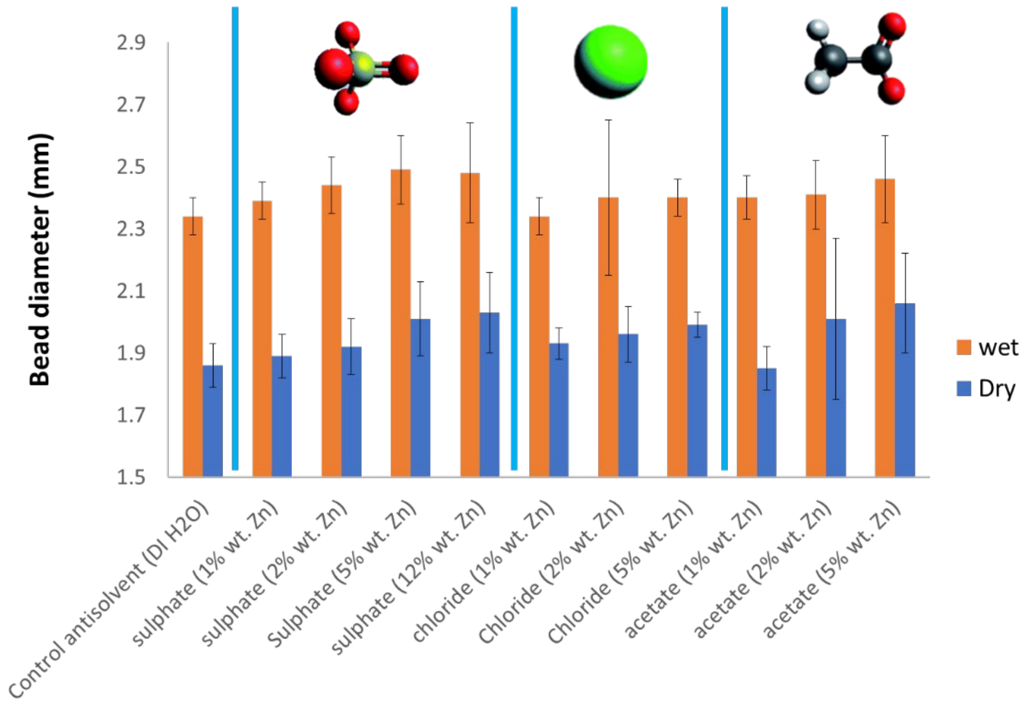

Figure S3 Average diameters of cellulose acetate beads produced using 15% wt. cellulose acetate solution in DMSO, using deionised water as a control and various aqueous zinc solutions as antisolvent. Beads were dried at 60 °C using a drying-cupboard over 3 days, until subsequent weighing of beads showed no further mass loss.

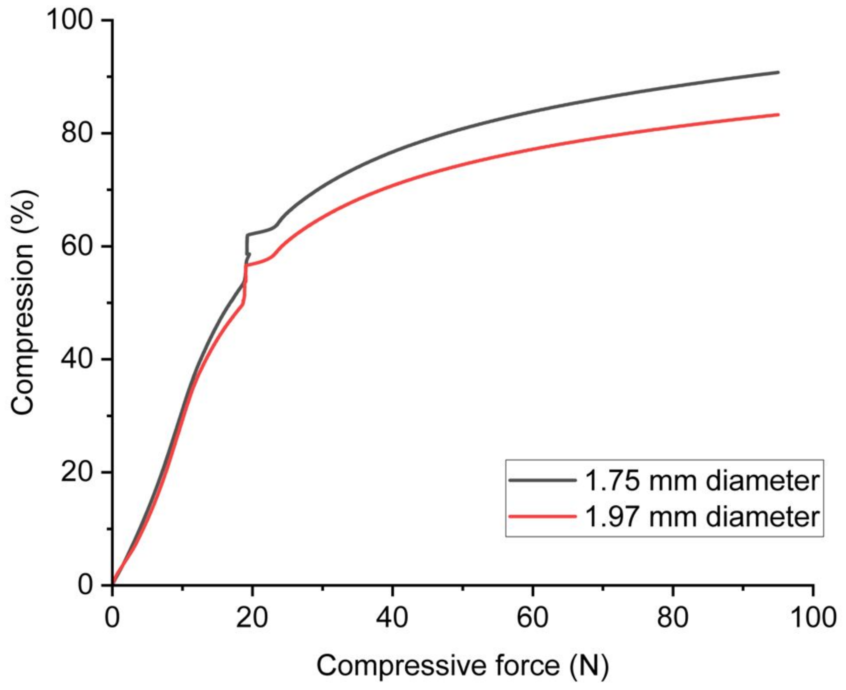

Figure S4. Compression testing of two cellulose acetate beads using an Instron 3369 with 100N load cell at 0.1 mm/min compression rate
